# FLRT2 mediates chondrogenesis of nasal septal cartilage and mandibular condyle cartilage

**DOI:** 10.1515/med-2024-0902

**Published:** 2024-03-22

**Authors:** Tao Xie, Fangyong Zhu, Renyi Cheng, Jing Gao, Yuchen Hong, Peishen Deng, Chaofeng Liu, Yanhua Xu

**Affiliations:** Department of Orthodontics, Kunming Medical University Affiliated Stomatological Hospital, Yunnan Key Laboratory of Stomatology, Kunming 650106, Yunnan, China; Department of Stomatology, Affiliated Hospital of Jiangnan University, Wuxi 214000, Jiangsu, China; Department of Stomatology, Panzhihua Central Hospital, Panzhihua 617000, Sichuan, China; Second Clinic, Kunming Medical University Affiliated Stomatological Hospital, Yunnan Key Laboratory of Stomatology, Kunming 650106, Yunnan, China; Party and Government Office, Kunming Medical University Affiliated Stomatological Hospital, Kunming 650106, Yunnan, China

**Keywords:** nasal septal cartilage, mandibular condyle cartilage, chondrogenesis, FLRT2

## Abstract

Nasal septal cartilages (NSCs) and mandibular condyle cartilages (MCCs) are two important cartilages for craniomaxillofacial development. However, the role of FLRT2 in the formation of NSCs and MCCs remains undiscovered. NSCs and MCCs were used for immunocytochemistry staining of collagen II, toluidine blue staining, and alcian blue staining. Quantitative reverse transcription‑PCR and western blot were used to detect mRNA and protein expressions of FLRT2, N-cadherin, collagen II, aggrecan, and SOX9. Cell proliferation of MCCs and NSCs was tested by 3-(4,5-dimethylthiazol-2-yl)-2,5-diphenyltetrazolium bromide assay and cell counting kit‑8 assay. Cell migration of MCCs and NSCs was examined by wound healing assay and Transwell. Chondrogenesis of MCCs and NSCs were similar in morphological characteristics, while different in cell proliferation, migration, and extracellular matrix. FLRT2 promotes the proliferation and migration of NSCs. There were up-regulation of N-cadherin and down-regulation of collagen II, aggrecan, and SOX9 in NSC with knock down FLRT2. The current study, as demonstrated by Xie et al., reveals that FLRT2 overexpression in Sprague-Dawley neonatal rats promotes the proliferation and migration of NSCs and MCCs, decreases N-cadherin while increases collagen II, aggrecan, and SOX9 in NSC and MCCs. Altogether, FLRT2 mediates chondrogenesis of NSCs and MCCs.

## 1 Introduction

Nasal septal cartilage (NSC) and mandibular condyle cartilage (MCC) are two important cartilages for craniomaxillofacial development. During embryonic development, NSC and MCC are neural crest cells that migrate to the frontonasal process and the first branchial arch, respectively, and then differentiate to form [1], the NSC eventually develops into primary cartilage, which is mainly composed of hyaline cartilage [2]. NSC is quadrangular hyaline cartilage which serves as a strut at the midline to support the lateral structures of the nose [3].

However, MCC eventually develop into secondary cartilage, which is mainly composed of fibrocartilage [4], its histologic structure, including articular or growth plate cartilage, is distinct from that of primary cartilage [5,6]. MCC acts as articular cartilage and growth plate cartilage as the template for longitudinal growth of mandibular ramous by endochondral ossification [7]. The differentiation of NSC and MCC into two different cartilage cells is closely related to the microenvironment of neural crest cells in these two cartilage sites [8].

During craniofacial development, FLRT2 plays an important role in mediating neural crest cell migration, cartilage formation, and epithelial–mesenchymal stem cell interaction; additionally, FLRT2 expression in the nasal septum region during embryonic development is higher than that of the mandibular primordium [9]; moreover, FLRT2 is highly expressed before the cranial neural crest cells migrate to the developing facial midpoint as well as during the process of cartilage formation [10].

Therefore, we propose that FLRT2 is involved in the formation of NSC and MCC. Current study demonstrated that overexpression of FLRT2 in Sprague-Dawley (SD) neonatal rats is related to the proliferation, migration, and cartilage differentiation of MCC and NSC, which lays a theoretical foundation for the formation of NSC and MCC, and also provides a theoretical reference for the prevention and correction of maxillofacial malformations.

## 2 Materials and methods

### 2.1 Experimental animals

NSCs and MCCs of SD neonatal rat nasal septum and condyle cartilage were isolated, washed by cold phosphate-buffered saline (PBS) at pH 7.4, and then used for the subsequent experimentations. Animal experiments were approved by the Animal Care and Use Committee of Kunming Medical University Affiliated Stomatological Hospital.

### 2.2 Cell culture and morphological characteristics of NSC and MCC

Isolated NSCs and MCCs were treated by 0.25% trypsin, ethylenediaminetetraacetic acid (EDTA), and 0.25 mg/mL collagenase type 2 (Life Technologies, Carlsbad, CA, USA) in 0.1 M PBS for 10 min at 37°C, to be dissociated into single cell suspensions. NSCs and MCCs were resuspended in Dulbecco’s Modified Eagle Medium containing 10% fetal bovine serum (FBS; Life Technologies, Carlsbad, CA, USA), 2.4 mg/mL *N*-(2-hydroxyethyl) piperazine-*N*′-(ethanesulfonic acid) (Nacalai Tesque, Kyoto, Japan), 0.2% sodium bicarbonate (Sigma-Aldrich, St Louis, MO, USA), and 100 U penicillin/streptomycin.

The morphological characteristics of NSCs and MCCs were observed by an inverted microscope (Olympus, Tokyo, Japan).

### 2.3 Immunocytochemistry (ICC)

The cultured NSCs and MCCs were used for ICC staining of collagen II at Days 5, 8, and 15, respectively. Briefly, after fixing with 4% paraformaldehyde (PFA) for 5 min, NSCs and MCCs were incubated with 0.25% Triton X-100 (Sigma-Aldrich, St. Louis, MO, USA) in PBS at room temperature for 10 min. Then, NSCs and MCCs were washed thrice by PBS, blocked with 1% PBS containing 2% bovine serum albumin (BSA) and 0.1% Tween-20 at room temperature for 1 h to block non-specific hybridization. Afterward, NSCs and MCCs were incubated with primary antibody against collagen II (ab34712; Abcam, Cambridge, UK) at 4°C overnight, and horseradish peroxidase (HRP)-labeled secondary antibody (ab288151; Abcam, Cambridge, UK) at room temperature for 1 h. Subsequently, NSCs and MCCs in each cover slip were immersed in a DAB detection kit (Dako North America, Inc., Carpinteria, CA, USA) until desired staining intensity developed, then counterstained in hematoxylin for 10 min. NSCs and MCCs were finally visualized by a light microscope (Olympus, Tokyo, Japan).

### 2.4 Alcian blue staining

The cultured NSCs and MCCs were used for alcian blue staining at Days 1, 3, 5, and 8, respectively. NSCs and MCCs were fixed in 4% PFA, stained with 0.5% alcian blue (Sigma‑Aldrich, St. Louis, MO, USA) in 3% acetic acid (pH 2.5) for 30 min. Subsequently, NSCs and MCCs were observed and images were captured by a light microscopy (Olympus, Tokyo, Japan). Image‑Pro Plus software (version 6.0; Media Cybernetics, Inc., Rockville, MD, USA) was used for the evaluation of the mean glycosaminoglycan (GAG) stain density in each image.

### 2.5 Toluidine blue staining

The cultured NSCs and MCCs (5 × 10^5^ cells/group) were used for toluidine blue staining at Days 5, 8, and 15, respectively. NSCs and MCCs were incubated at 65°C for 1 h, incubated in xylene (15 min, 4°C), and a concentration gradient of alcohol (5 min, 4°C). After being rinsed by double‑distilled water twice for 2 min each, NSCs and MCCs were incubated with toluidine blue staining solution (Solarbio Science & Technology Co., Ltd, Beijing, China) for 30 min at room temperature. NSCs and MCCs were then washed by double‑distilled water, sealed by neutral gum. At last, NSCs and MCCs were visualized under a light microscope (Olympus, Tokyo, Japan) to detect the integrated optical density in each image.

### 2.6 Quantitative reverse transcription‑PCR (qRT‑PCR)

Total RNA was isolated from MCCs and NSCs by TRIzol® (Invitrogen, Thermo Fisher Scientific, Waltham, MA, USA). The concentration and purity of RNA were determined by Nanodrop ND-1000 spectrophotometer (Thermo Fisher Scientific, Waltham, MA, USA). Afterward, mRNA was reverse transcribed into cDNA by First Strand cDNA Synthesis Kit (Takara, Osaka, Japan). Then, qRT-PCR was conducted on 7900HT Fast Real-Time system (Applied Biosystems, Foster, CA, USA). Thermocycle conditions were 95°C for 10 min, 35 cycles of 95°C for 20 s, and 58°C for 1 min. Relative mRNA expressions of FLRT2, N-cadherin, collagen II, aggrecan, and SOX9 were quantified by 2^−ΔΔCT^ method. β-actin served as an endogenous control.

### 2.7 Western blot

Total protein was extracted from MCCs and NSCs by radioimmunoprecipitation assay buffer (Sigma-Aldrich, St Louis, MO, USA) containing phenyl methane sulfonyl fluoride and protease inhibitor mixture (Roche Diagnostics, Indianapolis, IN, USA). The concentration of protein was determined by a bicinchoninic acid kit (Thermo Fisher Scientific, Waltham, MA, USA). Thereafter, protein sample was separated by 10% sodium dodecyl sulfate-polyacrylamide gel electrophoresis, followed by transferring onto poly-vinylidene fluoride (PVDF) membranes (Millipore, Billerica, MA, USA). Then, the PVDF membranes were subjected to blocking with 5% BSA (Beyotime, Shanghai, China), followed by incubation with primary antibodies against FLRT2, SOX9, and N-cadherin at 4°C overnight and HRP-conjugated secondary antibody at room temperature for 1 h, successively. Protein bands were visualized by the enhanced chemiluminescence (Bio-Rad, Hercules, CA, USA). Protein band intensities were analyzed by Image Lab™ (version 4.0; Bio‑Rad, Hercules, CA, USA). β-actin served as an endogenous control.

### 2.8 Knock down and overexpression of FLRT2

Lentiviral vector was used to knock down and overexpress FLRT2 gene. To achieve changes in the expression of FLRT2, NSCs (3 × 10^5^ cells/well) were plated into a 6-well plate, and transfected with FLRT2 short hairpin (sh)RNA (Sangon Biotech, Shanghai, China) or a nonsense strand NC by Lipofectamine® 2000 (Invitrogen, Thermo Fisher Scientific, Waltham, MA, USA) to knockdown FLRT2.

The full length of FLRT2 open reading frame was amplified from MCCs and NSCs cDNA, then ligated into pcDNA3.1 (Shaanxi Yuanbang Biotech, Shaanxi, China). For overexpression of FLRT2, MCCs and NSCs (3 × 10^5^ cells/well) were plated into a 6-well plate, and transfected with 2 μg pcDNA3.1‑FLRT2 or pcDNA3.1 with Lipofectamine® 2000 (Invitrogen, Thermo Fisher Scientific, Waltham, MA, USA) to overexpress FLRT2.

The transfection efficiencies of the shRNA and overexpression constructs were determined by qRT‑PCR and western blot at 48 h after transfection.

### 2.9 Determination of cell proliferation

#### 2.9.1 3-(4,5-Dimethylthiazol-2-yl)-2,5-diphenyltetrazolium bromide (MTT) assay

Briefly, cell proliferation of MCCs and NSCs from Days 1–8 as well as the effects of FLRT2-shRNA on the cell proliferation of NSC from Days 1–6 was examined by MTT assay (Sigma-Aldrich, St Louis, MO, USA). Cells (2 × 10^4^ cells/well) were seeded into 96-well plates, cultured in an incubator with humidified atmosphere containing 5% CO_2_ at 37°C for 24 h. Afterward, cells were added with 20 μL of MTT solution, and cultured in an incubator with humidified atmosphere containing 5% CO_2_ at 37°C for 4 h. Thereafter, the above mixture was added with 200 μL of dimethyl sulfoxide to dissolve the formazan crystals, and incubated for 15 min at 37°C. Optical density value of each sample was examined at 490 nm by a Microplate Reader (Bio‑Rad, Hercules, CA, USA).

#### 2.9.2 Cell counting kit (CCK)-8 assay

CCK‑8 (Dojindo Molecular Technologies, Gaithersburg, MD, USA) assay was used for the determination of the effects of FLRT2 overexpression on the cell proliferation of MCCs and NSCs (2 × 10^4^ cells/well) in 96-well plates at Days 1, 3, and 5, respectively. Briefly, cell medium was replaced by medium containing 10% CCK‑8, then MCCs and NSCs were incubated at 37°C for 2 h. The absorbance at 450 nm was determined by a Microplate Reader (Bio‑Rad, Hercules, CA, USA).

### 2.10 Determination of cell migration

#### 2.10.1 Wound healing assay

The migration ability of MCCs and NSCs was detected by the wound healing assay. In brief, 1 × 10^7^ MCCs and NSCs were seeded into 6-well plates and grown to 100% confluence. Next, a wound area was made at the center of each well by a 10 μL pipette tip. The medium was replaced by serum-free medium and incubated in an incubator with humidified atmosphere containing 5% CO_2_ at 37°C for 30 h. At 0 and 24 h, images of the wound area were captured. The percentage of migrated area was counted.

#### 2.10.2 Transwell assay

The migration capacity of MCCs and NSCs was detected by an 8 μm pore size Boyden chamber (Millipore, Billerica, MA, USA). MCCs and NSCs (5 × 10^3^ cells/well) were seeded into the upper Transwell chamber followed with the addition of serum-free culture medium, while culture medium containing 20% FBS was added into the lower Transwell chamber. Next, cells were cultured in an incubator with humidified atmosphere containing 5% CO_2_ at 37°C for 16 and 24 h, respectively. The migrated cells were fixed with 4% paraformaldehyde for 10 min, then stained with 0.1% crystal violet for 15 min at room temperature. A microscope (Olympus, Tokyo, Japan) was used for counting the migrated MCC and NSC.

### 2.11 Statistical analysis

Spss17.0 software was used to perform statistical analysis. Each experiment was repeated for at least thrice. One-way analysis of variance and independent *t*-test were used for statistical analysis. The results are expressed as mean ± standard deviation. *p* < 0.05 indicates a significant statistical difference.

## 3 Results

### 3.1 Chondrogenesis of MCCs and NSCs were similar in morphological characteristics

**Figure 1:**
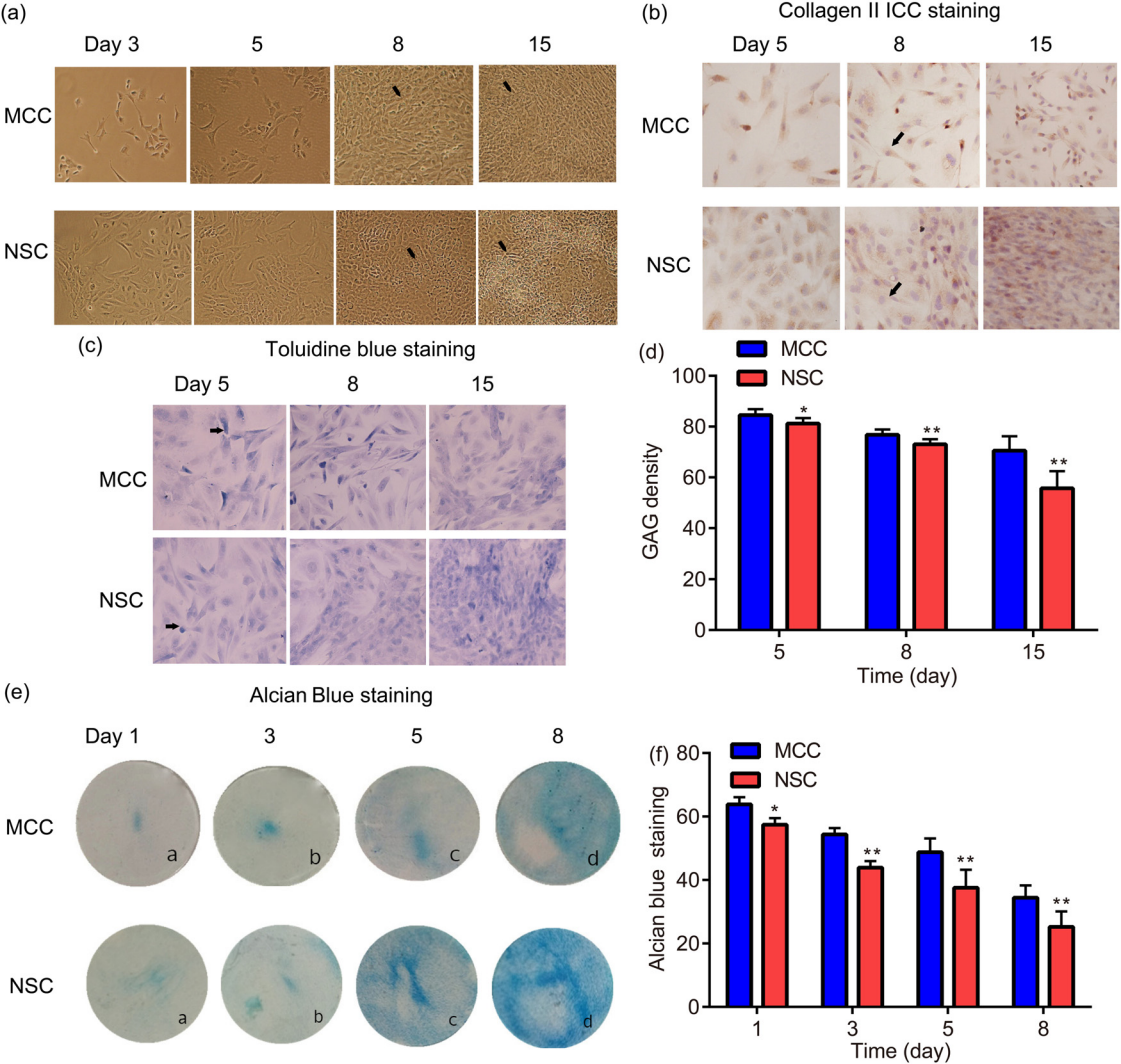
Chondrogenesis of MCCs and NSCs were similar in morphological characteristics. An inverted microscope was used to capture the freshly inoculated primary MCCs and NSCs, arrow heads indicated cells (a). ICC staining was used to detect collagen II secretion from NSCs and MCCs, arrow heads indicated collagen II (b). Toluidine blue staining was used to indicate GAG density in NSCs and MCCs, arrow heads indicated GAG (c) and (d). Alcian blue staining was applied to examine the extracellular matrix content of NSCs and MCCs (e) and (f). * *p* < 0.05, ** *p* < 0.01, vs MCCs.

Observed under an inverted microscope (Figure 1a), the freshly inoculated primary MCCs are larger than NSCs. On Day 0, the cell bodies are round, clear, and refractive, they begin to adhere to the wall about 12 h after inoculation; on Day 3, MCCs gradually expanded, mostly star-shaped, polygonal, and some spindle-shaped, adjacent cells were connected to each other by cytoplasmic protrusions, and cell bodies stretched; on Day 5, MCCs grow up to cover the bottom of the bottle, cell bodies are plump, the cytoplasm is uniform, the nucleoli are clear, cells are closely arranged with each other, and the local cells overlap; on Day 15, obvious cell aggregation can be seen in the colony-like growth area and the multi-layer growth area.

In addition, on Day 0, the newly inoculated primary NSCs were round, the cell size was relatively uniform, suspended, and the refraction was strong. NSCs adhered to the wall in 12 h, most of the adherence process was completed within 24 h; on Day 3, NSCs began to expand gradually, with increased volume and irregular triangle or polygon morphology; on Day 8, NSCs were connected by protrusions and formed a single layer, NSCs in many areas overlapped and grew, showing a typical “paving stone-like” structure; on Day 15, NSCs obviously aggregated and condensed, white “chondral-like” structures were visible.

The ICC staining exhibited that both MCCs and NSCs synthesize and secrete collagen II and the amount of NSCs is more than that of the MCCs (Figure 1b). Toluidine blue staining indicated that NSCs exerted lower GAG density compared to MCCs (Figure 1c and d). Alcian blue staining demonstrated that there are differences in the extracellular matrix content between NSCs and MCCs (Figure 1e and f), indicating inherent variances in the functional dynamics between MCCs and NSCs.

### 3.2 Chondrogenesis of MCCs and NSCs were different in cell proliferation, migration, and extracellular matrix

**Figure 2:**
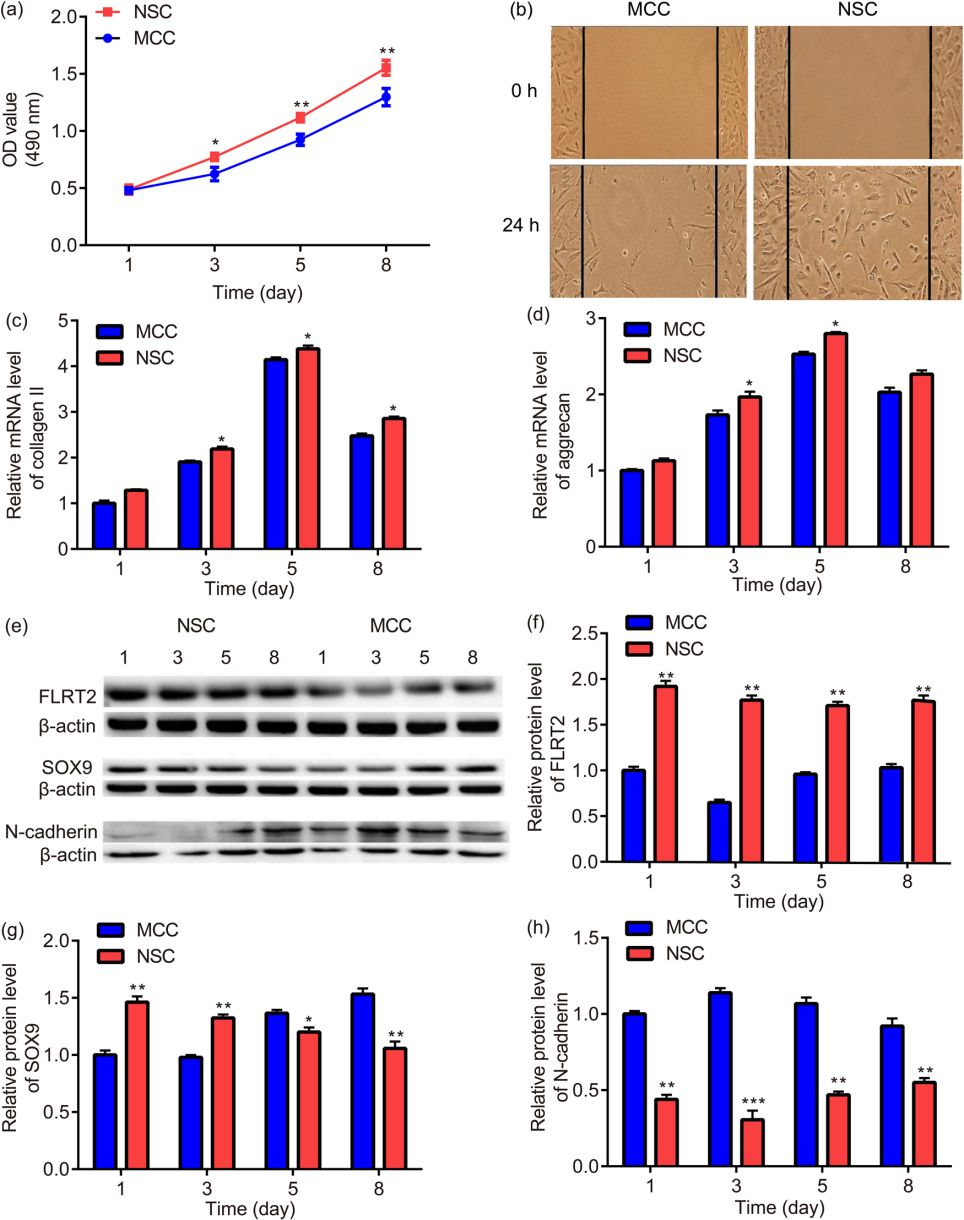
Chondrogenesis of MCCs and NSCs were different in cell proliferation, migration, and extracellular matrix: MTT assay was used for the examination of MCCs and NSCs cell proliferation (a). The scratch experiment was applied for the detection of MCCs and NSCs cell migration (b). RT-PCR was used to detect the mRNA expressions of type Ⅱ collagen (c) and aggrecan (d) in MCCs and NSCs. Western blot was used to detect the protein expressions of FLRT2 (e) and (f), SOX9 (e and g), and N-cadherin (e and h) in MCCs and NSCs. * *p* < 0.05, ** *p* < 0.01, *** *p* < 0.001, vs MCCs.

The proliferation trend of MCCs and NSCs is roughly the same, while the cell proliferation of NSCs was stronger than that of MCCs (Figure 2a); moreover, the cell viability of MCCs and NSCs is not changed (data not shown).

In the scratch experiment, there were more NSCs than MCCs in the center of the scratch, indicating that the migration ability of NSCs was higher than that of MCCs (Figure 2b).

RT-PCR was used to detect the mRNA expressions of type Ⅱ collagen and aggrecan in MCCs and NSCs. It was found that the MCCs and NSCs had expression of type Ⅱ collagen and aggrecan on Day 1, which increased on Day 3, and peaked on Day 5; moreover, the expression of type Ⅱ collagen and aggrecan in NSCs was statistically stronger than that of MCCs (Figure 2c and d).

The protein expressions of multifactors in MCCs and NSCs were also different. Western blot indicated that there was higher expression of FLRT2 in NSCs than in MCCs (Figure 2e and f), also, there was higher expression of SOX9 in NSCs than in MCCs at Days 1–3; however, there was higher expression of SOX9 in MCCs after the third day (Figure 2e and g), while there was lower expression of N-cadherin in NSCs than in MCCs (Figure 2e and h).

### 3.3 FLRT2 promotes the proliferation and migration of NSCs

**Figure 3:**
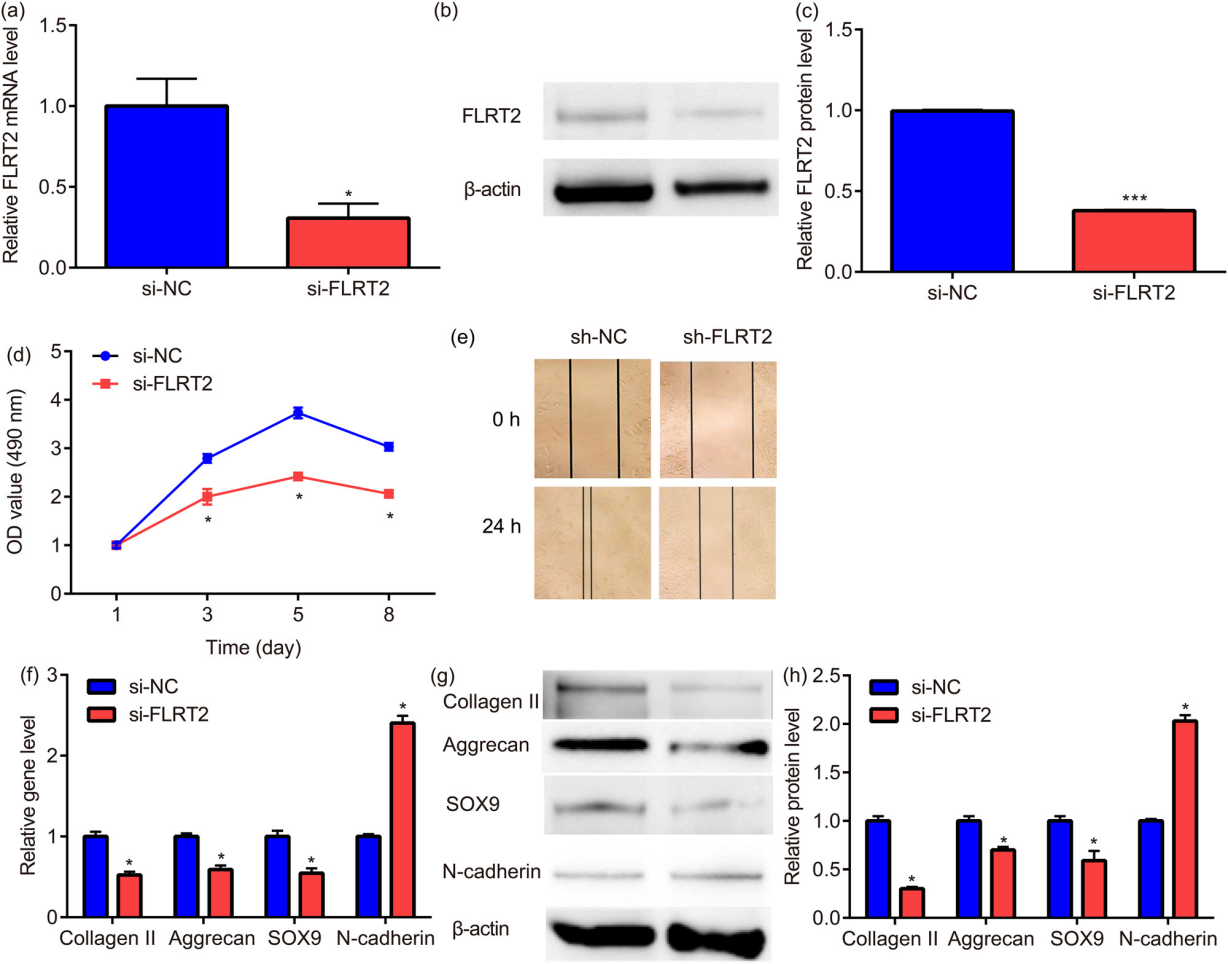
FLRT2 knockdown inhibits the proliferation, migration, and matrix synthesis/secretion of NSCs. The FLRT2 gene knockdown efficiency in NSCs was verified by RT-PCR and western blot (a)–(c). The NSC cell proliferation was evaluated by MTT assay (d). The NSC cell migration was determined by scratch assay (e). The collagen II, aggrecan, SOX9, and N-cadherin mRNA and protein expressions of NSCs were assessed by RT-PCR and western blot (f)–(h). * *p* < 0.05, vs MCC.

The effect of FLRT2 knockdown in NSCs was verified by RT-PCR and western blot. The FLRT2 gene knockdown efficiency reached 68% (Figure 3a–c), indicating the establishment of NSCs with knocking down of FLRT2.

The proliferation trend of MCCs and NSCs was roughly the same. The number of cells began to increase rapidly on Day 2 (1.66 ± 0.09 vs 1.46 ± 0.07), the growth rate slowed down on Day 3 (2.79 ± 0.12 vs 2.00 ± 0.16), and the cell growth rate increased again on Day 4 (2.91 ± 0.12 vs 2.11 ± 0.06), and reached a peak on Day 5 (3.73 ± 0.11 vs 2.42 ± 0.07). Comparing with sh-NC group, there was no statistical difference in sh-FLRT2 group from Day 1 to Day 2; however, there was a statistical reduction from Day 3 to Day 8 (Figure 3d), indicating that FLRT2 promotes the proliferation of NSCs. In addition, the cell viability of NSCs is not changed (data not shown).

The width of the scratch is measured at 0 and 24 h, showing that the migration in each group increased with time; compared with sh-NC, the migration of NSCs transfected with sh-FLRT2 was significantly reduced (Figure 3e), suggesting that FLRT2 promotes the migration of NSCs.

### 3.4 Up-regulation of N-cadherin and down-regulation of collagen II, aggrecan, and SOX9 in NSC with knock down FLRT2

Compared with sh-NC group, N-cadherin mRNA and protein expression of NSCs transfected with sh-FLRT2 was significantly increased, while collagen II, aggrecan, and SOX9 mRNA and protein expressions of NSCs transfected with sh-FLRT2 were significantly decreased (Figure 3f–h).

### 3.5 Overexpression of FLRT2 promotes the proliferation and migration of NSCs and MCCs

**Figure 4:**
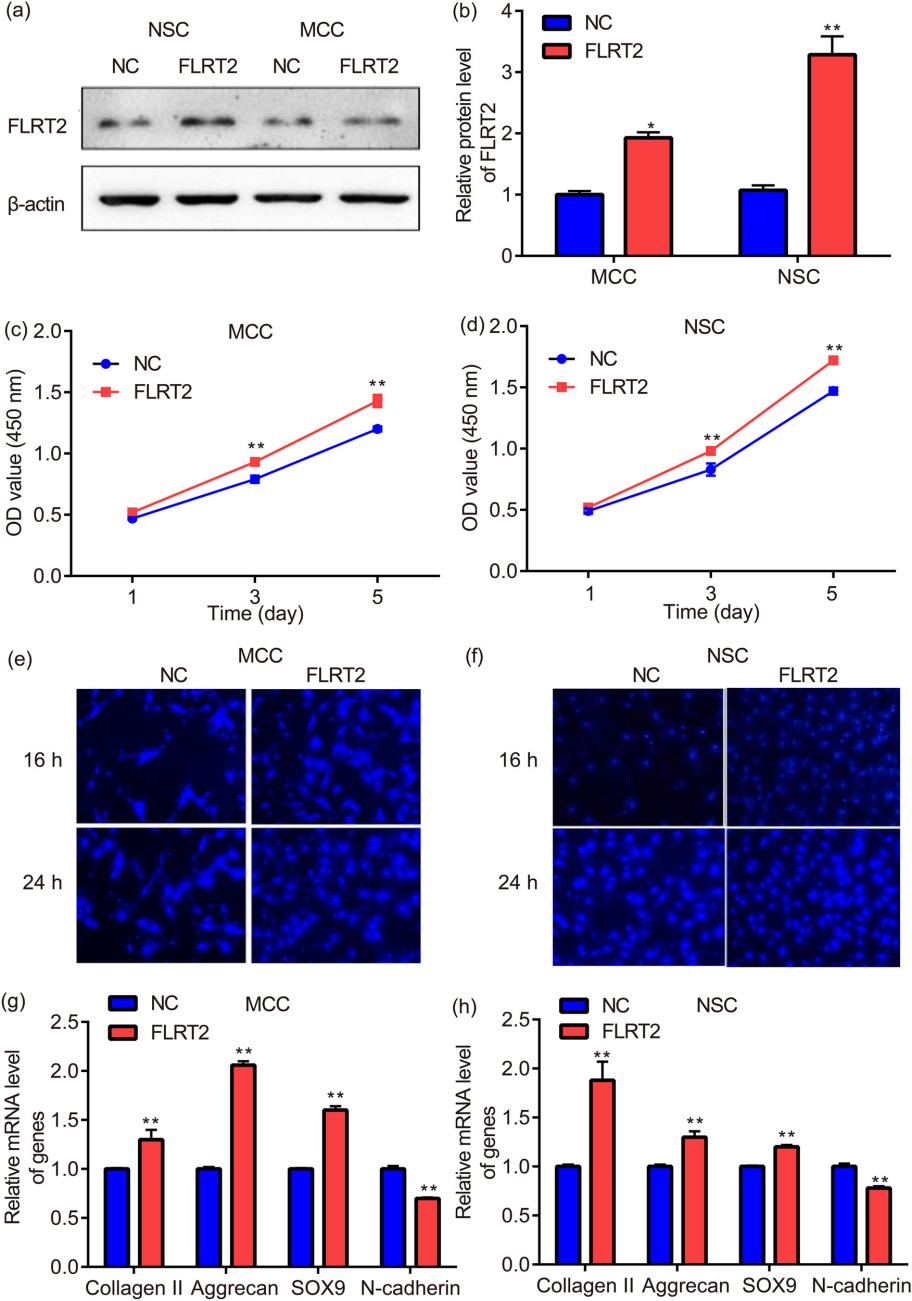
FLRT2 overexpression promotes the proliferation, migration, and matrix synthesis/secretion of NSCs and MCCs. Western blot was used to verify whether FLRT2 was overexpressed in NSCs and MCCs (a) and (b). CCK-8 assay was utilized for the detection of NSC and MCC cell proliferation (c) and (d). Transwell assay was applied for the evaluation of NSC and MCC cell migration (e) and (f). RT-PCR was used for the assessment of collagen II, aggrecan, SOX9, and N-cadherin mRNA expressions in NSCs and MCCs (g) and (h). * *p* < 0.05, ** *p* < 0.01, vs MCC.

The chondrocytes were subjected to western blot to verify whether FLRT2 was overexpressed, results showed that FLRT2 protein synthesis in NSCs and MCCs increased; moreover, the increase in NSCs was more obvious than that in MCCs (Figure 4a and b).

The proliferation trend is almost the same for NSCs and MCCs; moreover, the increase of NSCs was more obvious than that of MCCs (Figure 4c and d). Additionally, the cell viability of MCCs and NSCs is not changed (data not shown).

Overexpression of FLRT2 promotes cell migration of NSCs and MCCs at 16 and 24 h (Figure 4e and f).

### 3.6 Overexpression of FLRT2 decreased N-cadherin while increased collagen II, aggrecan, and SOX9 in NSCs and MCCs

Compared with NC group, N-cadherin mRNA expression of NSCs and MCCs transfected with FLRT2 was significantly decreased, while collagen II, aggrecan, and SOX9 mRNA expressions of NSCs and MCCs transfected with FLRT2 was significantly increased (Figure 4g and h).

## 4 Discussion

There are different morphological characteristics of MCCs and NSCs. At Day 0, the inoculated primary MCCs and NSCs are round, suspended in the culture medium, the cell body is clear and refractive, and began to adhere to the wall about 12 h after inoculation. At Day 1, most of the cells adhered to the wall, the morphology of MCCs and NSCs were triangular or fusiform, the cell expansion volume increased, some cells gradually protruded outward, and the cell morphology became polygonal (data not shown). At Day 3, cells were long triangles, all attached to the wall, and cell number began to increase. At Day 5, cell number increased significantly, the cells formed triangles, and part of the cells overlapped and connected in a porous shape. At Days 8–15, the cells were all overgrown in the flask and they were full of paving stones.

The chondrogenic cells are surrounded by a large amount of extracellular matrix, so that they can promote or inhibit the growth of cartilage [11]. Cell proliferation and matrix synthesis/secretion of NSCs occur in the differentiated chondrogenic cells at the deep layer of cartilage [12], while the secondary cell proliferation of MCCs occurs in the undifferentiated mesenchymal cells on the surface of the cartilage tissue, and the matrix synthesis occurs in the deep differentiated chondrocytes [13]. When factors are isolated, NSCs are found to be less affected by local mechanical factors; the deep differentiated chondrocytes are not surrounded by the extraneous chondrocyte matrix, so MCCs are susceptible to local factors. The difference in the synthesis of extracellular matrix between NSCs and MCCs at each stage of growth leads to their different responses to local biomechanical factors.

Type II collagen provides cartilage tissue with properties of tensile strength and stretching ability [14,15]. Combined with the cartilage collagen network, the cartilage has properties such as elasticity and tensile pressure [16,17]. The NSCs synthesize and secrete collagen and the amount is more than that of the MCCs. Therefore, the NSCs may have stronger anti-pressure properties than MCCs, so as to better maintain the stability of the NSCs and support the middle structure of the surface [18].

Extracellular matrix has important functions, especially in the process of chondrocyte proliferation, migration, and agglutination [19]. Gong et al. [9,10] found that during the development of the craniofacial region, FLRT2 is highly expressed before the cranial neural crest cells migrate to the developing facial midpoint and during the entire process of cartilage formation, with the most obvious in cartilage area. They speculate that FLRT2 plays a key regulatory role in the development of NSCs. Xu et al. [20] established the NSC progenitor cell line (ATDC5) model, which shows that FLRT2 promotes the proliferation of chondrocytes and inhibit chondrocyte adhesion; meanwhile, extracellular matrix such as GAG and N-cadherin changes.

Herein, compared with sh-NC, the cell proliferation in the sh-FLRT2 group was inhibited, while in the FLRT2 group was enhanced, indicating that FLRT2 promotes the proliferation of chondrocyte progenitor cells. In the sh-FLRT2 group, N-cadherin is down-regulated, while GAGs and type II collagen are up-regulated. Current study suggests that FLRT2 has a regulatory effect on cell proliferation during cartilage development that is inhibiting cell adhesion as a previous study report [21].

Fibroblast growth factor (FGF) signals promote the proliferation of chondrocytes in the process of cartilage formation. Fibroblast Growth Factor Receptor 2 (FGFR2), as a receptor of FGF signaling, exerts a close effect on FLRT2 [22]. FGFR2 is a transmembrane protein, including an extracellular, transmembrane, and intracellular tyrosine kinase structure [23,24]. Although the action mechanism between FLRT family and FGFR remains divergent, some scholars have found that FLRT2 positively feedback regulated FGFR2, and the increase in FLRT2 up-regulated FGFR2 expression [25]. The extracellular and intracellular structures of FLRT2 interact with the extracellular and intracellular regions of FGFR2, respectively [26].

Herein, FLRT2 mainly promotes cell proliferation and migration; moreover, the proliferation and migration ability of MCCs and NSCs are basically the same, which in NSCs is significantly higher than that in MCCs, suggesting that FLRT2 has a stronger regulatory effect on NSCs. The results of current study are consistent with previous studies on ATCD5 cells [20].

Furthermore, the changing of trend over time for type Ⅱ collagen and aggrecan are in consistent with the proliferation trend of MCCs and NSCs, peaked on Day 5 and dropped after that, which might be attributable to phenomenon that the chondrocytes proliferate and form typical cartilaginous nodules on Day 5 of cultures [27].

By determination of mRNA expression of N-cadherin, AGG, Sox-9, and col II, it is found that N-cadherin decreases, while the rests were up-regulated with the expression of FLRT2. Therefore, current study proved that FLRT2 mainly regulates the function of extracellular matrix, inhibits the aggregation and adhesion between cells, and controls the proliferation and migration.

In conclusion, by comparison of the biological characteristics of MCCs and NSCs, it was found that the migration and proliferation of NSCs were stronger than MCCs. In NSCs, collagen II and aggrecan expression were higher, while N-cadherin expression was lower than MCCs. Although they have many differences, they are both regulated by FLRT2. FLRT2 plays a role in mediating cellular events (cell–cell, cell–matrix interactions, and cell proliferation) during chondrogenesis of NSCs and MCCs.

However, there is a limitation in current study, for the overexpression experiments, since the observed increase in FLRT2 expression level is relatively modest, it is necessary to optimize the experimental conditions in the future work.
